# Combined microRNA and ER expression: a new classifier for familial and sporadic breast cancer patients

**DOI:** 10.1186/s12967-014-0319-6

**Published:** 2014-11-19

**Authors:** Katia Danza, Simona De Summa, Brunella Pilato, Massimo Carella, Orazio Palumbo, Ondina Popescu, Angelo Paradiso, Rosamaria Pinto, Stefania Tommasi

**Affiliations:** Molecular Genetics Laboratory, IRCCS, Istituto Tumori “Giovanni Paolo II”, v.le Orazio Flacco 65, 70124 Bari, Italy; Medical Genetics Unit, IRCCS Casa Sollievo della Sofferenza – San Giovanni Rotondo (FG), Bari, Italy; Anatomopathology Unit, IRCCS, Istituto Tumori “Giovanni Paolo II”, Bari, Italy; Experimental Medical Oncology Unit, IRCCS, Istituto Tumori “Giovanni Paolo II”, Bari, Italy

**Keywords:** Familial breast cancer, miRNA, TNBC, BRCA

## Abstract

**Background:**

The role of miRNAs in familial breast cancer (fBC) is poorly investigated as also in the BRCA-like tumors. To identify a specific miRNA expression pattern which could allow a better fBC classification not only based on clinico-pathological and immunophenotypical parameters we analyzed miRNA profile in familial and sporadic samples. Moreover since BRCA1 tumors and sporadic triple negative (TN) breast tumors share similarities regarding clinical outcomes and some histological characteristics, we focused on TN and not TN cases.

**Methods:**

The sample set included fresh frozen tissue samples, including 39 female fBCs (19 BRCA-related and 20 BRCAX) and 12 male fBC (BRCAX). Moreover, we considered TN and non TN (NTN), 21 BRCA-related and 27 sporadic BCs. MiRNA profiling was performed through GeneChip miRNA v.1.0 Array (Affymetrix). ANOVA, hierarchical and consensus clustering analyses allowed identification of pattern of expression of miRNAs and pathway enrichment analysis, considering validated target genes, was carried out to achieve a deeper biological understanding.

**Results:**

ANOVA test led to the identification of 53 deregulated miRNAs; hierarchical and consensus clustering of female fBCs (fFBCs) and male fBCs (fMBCs) highlighted the presence of 3 sample clusters named FBC1, FBC2 and FBC3. We found a correlation between ER-status and the three sample clusters. The three clusters are distinct by a different expression of two clusters of miRNAs (CLU1 and CLU2), which resulted to be different in targeted pathways. In particular, CLU1 targets cellular pathways and CLU2 is involved in epigenetic activities. Considering TN and NTN BRCA-related and sporadic tumors, a hierarchical clustering identified two clusters of miRNAs, which were not so different from CLU1 and CLU2, both in miRNA content and targeted pathways.

**Conclusions:**

Our results highlighted the importance of miRNA regulation to better clarify similarities and differences between familial and sporadic BC groups.

## Introduction

Breast cancer (BC) is a very heterogeneous disease. Patients with a family history of BC (5-7%) account for germline mutations in the high susceptibility genes BRCA1/2 (25%), while 20-25% of fBC can be attributed to other high-moderate-low susceptibility genes [1]. Moreover for about the 50% of familial BC (fBC), that show no mutation in any of these genes, it has been proposed a polygenic model in which the susceptibility is conferred by the action of several low-penetrance loci [1]. The genetic alterations associated with breast carcinogenesis are mostly studied; on the contrary, epigenetic alterations in fBC is a new field of interest. The concept of epigenetics refers to changes in gene activity that does not involve variations in the primary DNA sequence [2,3]. The most widely studied class of non coding RNAs are the microRNAs (miRNAs) [4]. They play an important role in post-transcriptional gene silencing regulating gene expression by targeting RNA degradation or translational inhibition through interaction with the 3′ untranslated region (UTR) of the target mRNA. Although there are several reports on the miRNA expression profile in BC and in different types of BC [5-8], results are often controversial, leaving the question open as to whether miRNA profiling can be used or not to differentiate BC patients. Moreover very little has been reported about the role of the miRNAs in the subgroups of fBC or in the BRCA-like tumors.

Recently, studying some miRNAs related to BRCA genes in fBC, we highlighted the involvement of miR-17, miR-21, let-7a in familial compared to sporadic BC and further their higher expression associated with BRCA1/2 mutations [9].

Tanic et al. identified a 17 miRNA signature in fBC when comparing to normal breast tissue showing that many of the deregulated miRNAs were involved in the MAPK signaling pathway in both familial and sporadic tumors [10]. However, Tanic et al. further explored the tumor heterogeneity of BC patients without BRCA1 and BRCA2 mutations (BRCAX patients). They highlighted four different subgroups (BRCAX-A, −B, −C and -D), characterized by 3 specific miRNA clusters and histopathological features [11].

Recently, 15 miRNAs, able to differentiate among the four groups (BRCA1, BRCA2, sporadic BC and BRCAX), have been identified [12]. Each group was composed by 5 BC cases. The first three groups were associated with distinct clusters of hyper-expressed miRNAs compared to BRCAX where all these miRNAs were hypo-expressed. They also found specific miRNAs associated with ER, PR, and HER2/neu status, Ki 67 and phenotype [12].

However few data are still available on miRNA expression associated to different clinico-pathological features in fBCs and in subgroups of sporadic BCs.

Currently, BC classification and choice of treatment still depend on immunohistochemical (IHC) analysis of markers among which ER, PR and HER2 are fundamental to define Triple Negative BC (TNBC) tumors as ER-, PR- and HER2-. These last tumors seem to behave as BRCA1-mutated tumors [13,14]. Among the BRCA1-mutated breast tumors, the triple negative phenotype represents 70-80%. In particular, patients under 50 years old and with BRCA1 germline mutations have morphological features similar to those described for triple negative tumors. Studies on the role of miRNAs to stratify TNBC did not provide clear information. The first study totally focused on TNBC miRNA profiling was by Cascione et al. in 2013 [5]. Comparing primary TNBC and normal tissues, the miRNA profiling revealed 116 deregulated miRNAs, among which miR-106b, the cluster miR-17/92, miR-8 family, miR-21 and miR-155 were the most up-modulated while let-7b, let-7c, miR-126, miR-145 and miR-205 were the most down-modulated [5]. The miR-200 family is a known negative regulator of the epithelial-mesenchymal transition (EMT), through the direct targeting of Zeb1/Zeb2 [15]. This miRNA family appears to be one of the most interesting players in TNBC biology and it was previously described as up-modulated in BC where its over-expression was correlated with lymph node positivity and metastasis. The miR-205 exerts a clearer tumor-suppressive role. Iorio’s group described its down-modulation in TNBC, in particular in the claudin-low subgroup [16]. Besides miR-200 family and miR-205, it must also mention other tumor suppressor miRNAs particularly involved in TNBC as miR-203, miR-31, miR-34a; while TNBC oncomiR are miR-181a/b, miR-146 and miR-146b-5p and miR-182 [16].

In order to identify miRNA expression pattern which could support BC classification in familial BC, we analyzed miRNA expression in 51 familial sample patients. Moreover, to better classify TNBCs we compared miRNA expression of 21 BRCA carriers and 27 sporadic BC cases. The two main clusters highlighted by hierarchical analysis evidenced a driver role of ER in fBC. Furthermore, we demonstrated that BRCA-related and sporadic TNBC clustered together supporting the hypothesis of similar epigenetic regulation in these tumors.

## Materials and methods

### Sample set

**Figure 1:**
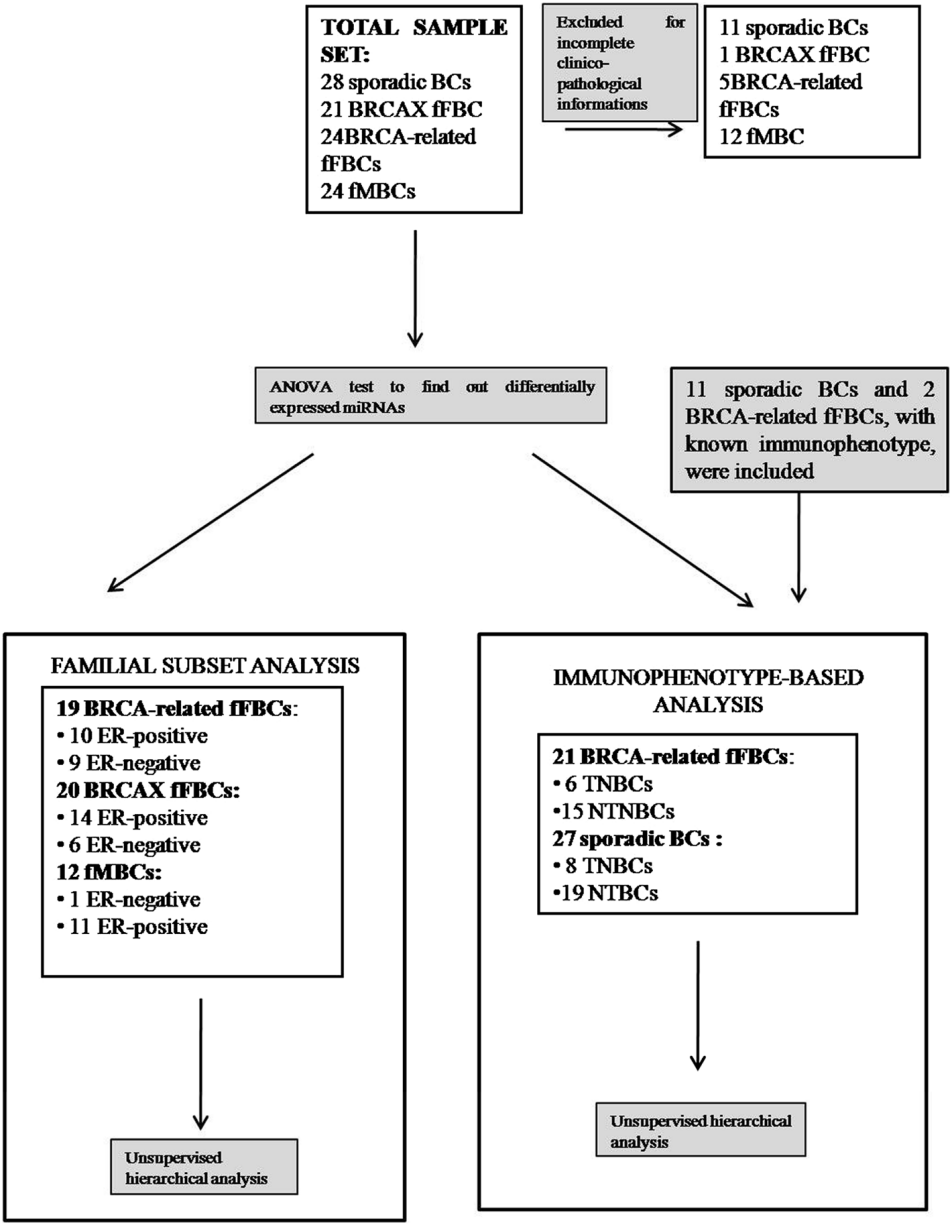
**Analysis workflow.** Features of the sample sets are described.

A set of 51 fBC patients (12 male and 39 female cases) was enrolled through the Genetic Counseling Program at the IRCCS, Istituto Tumori “Giovanni Paolo II” in Bari, Italy. Patients signed informed consent giving permission to use their pathological material. In detail, familial female BCs (fFBCs) included 19 BRCA-related, indicating with this term patients carrying germline deleterious mutations in BRCA1/2 genes, and 20 BRCAX. The term “BRCAX” is used to indicate those patients with familial BC carrying no mutation in BRCA1/2 genes. Familial male BCs (fMBCs) are only BRCAX. BRCA1/2 gene mutational status was evaluated through capillary sequencing on blood-extracted DNA. The analyses on TNBC and NTNBC have been performed in 21 BRCA-related and 27 sporadic BCs. The flowchart of the study is described in Figure 1.

### RNA extraction

RNA was extracted from fresh frozen cancer specimens containing at least 70% tumor cells and from normal tissues using the RNeasy Plus Mini Kit (Qiagen, Valencia CA) according to the manufacturer’s protocol. Concentrations were estimated with the ND-8000 Spectrophotometer (NanoDrop Technologies, Wilmington, DE).

### Microarray hybridization and data preprocessing

Five hundred ng of RNA of each sample were labelled by using the 3DNA Array Detection FlashTagTM RNA Labeling Kit according with manufacturer’s instruction and analyzed by the Gene Chip miRNA v. 1.0 Array (Affymetrix) which contains 46,228 probes comprising 7,815 probe sets and covers 71 organisms including 1100 human miRNAs derived from the Sanger miRBase and miRNA database v11 (April 15, 2008, http://microrna.sanger.ac.uk). First, poly (A) tailing was carried out at 37°C for 15 min in a volume of 15 ml reaction mix, which contained 1X Reaction Buffer, 1.5 ml MgCl_2_ [25 mM], 1 ml ATP Mix diluted 1:500 and 1 ml PAP enzyme. Second, Flash Tag Ligation was performed at room temperature for 30 min by adding 4 ml of 5X Flash Tag Ligation Mix Biotin and 2 ml T4 DNA Ligase into the 15 ml of reaction mix. To stop the reaction, 2.5 ml of Stop Solution was added. Each sample were hybridized on the array, washed and stained with the Affymetrix Fluidics Station 450 and scanned with the AffymetrixGeneChip Scanner 3000 7G using the Command Console software (Affymetrix).Raw data (.CEL files) were normalized through Robust Multi-array Average (RMA) method to remove systematic variations. Briefly, RMA corrects raw data for background using a formula which is based on a normal distribution and uses a linear model to estimate values on a log-scale. RMA normalization was performed using the “affy” package of the Bioconductor suite (http://www.bioconductor.org/) for the R statistical language (http://cran.r-project.org/). Microarray dataset has been deposited at the ArrayExpress database under the accession number E-MTAB-2705.

### Differential expression analysis

Normalized values were statistically analyzed with MeV software v.4.8.1 [17]. Differentially expressed miRNAs were detected through ANOVA, using 500 permutations. The unadjusted P-values were corrected for multiple hypotheses testing using Benjamini and Hochberg false discovery rate (FDR < 0.05) [18]. Data were considered to be statistical significant when p < 0.01.

### Class discovery methods

Unsupervised average-linkage hierarchical clustering using Pearson correlation was performed through “Hierarchical clustering” module of Gene Pattern suite [19]. “Consensus clustering” module was used for class discovery and clustering validation. Such an analysis was performed with KNN means algorithm with 2, 3, 4 and 5 centroids through 500 resampling iterations.

Gini correlation coefficient has been used to assess the optimal number of clusters. The Gini correlation coefficient has been borrowed from economics, sociology, physics, engineering, and informatics to solve a series of mathematic problems without having to hypothesize the form of data distribution [20]. The Gini correlation is more robust on non-normally distributed data and it is more stable for data containing outliers, compared with the correlation methods based on normal distributions. Moreover, it provides higher accuracy than correlation methods that only use rank information. “Delta Gini” was introduced to consider the differences in the inequality of edge weights between two networks and it was used to validate unsupervised hierarchical analysis.

### Pathway enrichment analysis

Pathway enrichment analysis was performed considering validated targets for each miRNAs in clusters. Results were obtained from Tarbase [21], miRtarbase [22] and miRecords [23]. We used these databases because they collected experimentally validated miRNA targets (e.g., reporter assay, western and northern blot, qRT-PCR). The resulting gene list for each cluster was submitted to DAVID 6.7 bioinformatic tool [24] in order to identify the targeted pathway, setting the threshold for FDR to 0.01 and considering enrichment in Gene Ontology (GO) terms.

## Results

### Unsupervised hierarchical clustering of familial breast tumors and ER status

**Figure 2:**
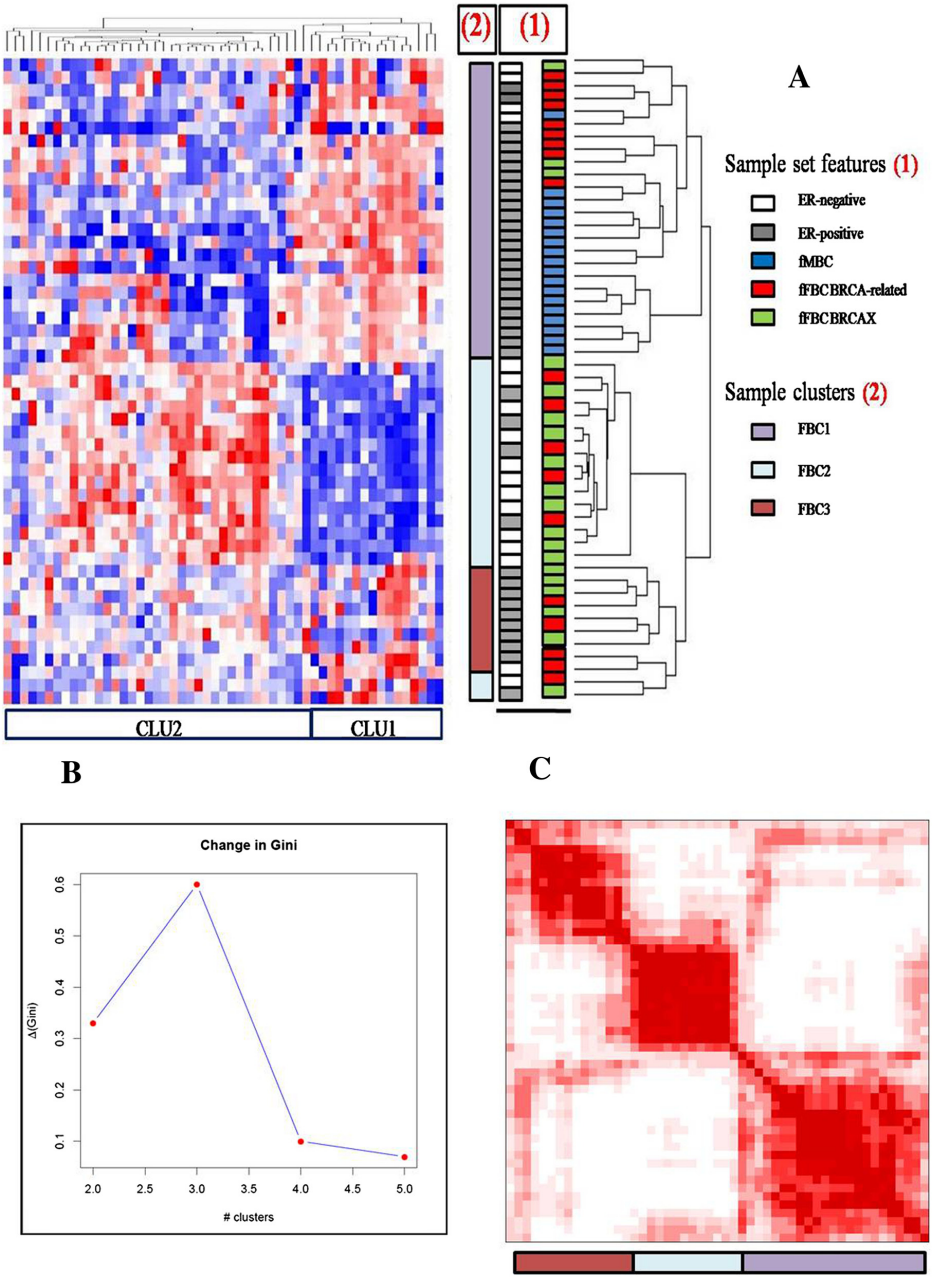
**Clustering analysis of familial breast tumors. A)** Unsupervised hierarchical clustering of fFBCs (BRCA-related and BRCAX) and FMBCs over 53 deregulated miRNAs (Pink indicates overexpressed miRNAs; blue indicates underexpressed miRNAs); **B)** Bootstrap analysis of the sample set by Consensus Clustering. Plot shows change in Gini correlation coefficient (ΔGn) with each additional group added, indicating that the optimal numbers of clusters is three; **C)** Red squares in the consensus matrix represent the subgroup of the sample set. fMBC: familial male breast cancer; fFBC: familial female breast cancer.

Our sample set included fFBCs (19 BRCA-related and 20 BRCAX), fMBC (12 BRCAX) and sporadic BCs (n = 27) (Figure 1). The ANOVA test performed among the four groups, identified 53 differentially expressed miRNAs (p < 0.01, FDR < 0.05) (Table 1). Unsupervised hierarchical clustering (Figure 2A) and consensus clustering (Figure 2B-C) in the fBC subset, including male and female cases, highlighted the presence of 3 sample clusters (named FBC1, FBC2, FBC3). In particular, in Figure 2B it could be observed the variation of Gini coefficient which stated as the optimal number for patient clusters as 3 because at this value the greatest value of ΔGini was reached, indicating the greatest inequality between them.

**Table 1:** **ANOVA test results from the comparison between male and female familial breast cancer and sporadic tumors**

	**fMBC**	**fFBC**			
	**BRCAX**	**BRCA-related**	**BRCAX**	**sporadic**	***P***
**hsa-miR-106b**	9.34 ± 0.57	5.88 ± 2.72	5.09 ± 2.48	5.69 ± 2.63	5.49E-05
**hsa-miR-1259**	2.15 ± 0.15	2.31 ± 0.15	2.47 ± 0.24	2.27 ± 0.18	8.93E-05
**hsa-miR-125a-3p**	6.01 ± 1.04	4.11 ± 1.22	3.76 ± 1.21	3.83 ± 1.36	1.00E-05
**hsa-miR-1271**	3.58 ± 0.81	3.01 ± 0.44	2.62 ± 0.30	2.73 ± 1.36	1.59E-05
**hsa-miR-1274a**	6.83 ± 1.78	3.73 ± 1.68	2.88 ± 0.98	3.65 ± 1.23	2.99E-10
**hsa-miR-1274b**	9.44 ± 1.38	5.78 ± 2.48	4.31 ± 2.04	5.69 ± 2.30	1.70E-07
**hsa-miR-128**	4.32 ± 1.56	2.95 ± 0.74	2.57 ± 0.33	2.80 ± 0.63	4.66E-07
**hsa-miR-140-5p**	3.13 ± 0.83	2.58 ± 0.46	2.30 ± 0.15	2.36 ± 0.20	1.83E-06
**hsa-miR-143-star**	4.03 ± 1.43	2.51 ± 0.38	2.53 ± 0.40	2.53 ± 0.42	5.70E-09
**hsa-miR-143**	10.12 ± 1.36	6.82 ± 2.58	6.08 ± 2.66	6.11 ± 2.87	8.81E-05
**hsa-miR-148a**	4.45 ± 1.62	2.83 ± 0.57	2.66 ± 0.55	3.11 ± 1.08	9.25E-06
**hsa-miR-148b**	3.34 ± 0.99	2.50 ± 0.23	2.46 ± 0.26	2.48 ± 0.34	1.53E-06
**hsa-miR-152**	7.76 ± 1.36	4.82 ± 1.84	4.07 ± 1.82	4.52 ± 1.82	1.11E-06
**hsa-miR-15a**	6.72 ± 0.87	3.95 ± 1.27	3.20 ± 1.05	3.88 ± 1.41	1.39E-10
**hsa-miR-17-star**	4.43 ± 0.68	3.12 ± 0.95	2.58 ± 0.44	2.70 ± 0.56	1.73E-10
**hsa-miR-181c-star**	3.10 ± 0.82	2.43 ± 0.28	2.51 ± 0.31	2.41 ± 0.24	2.69E-05
**hsa-miR-188-5p**	3.19 ± 0.43	2.55 ± 0.35	2.50 ± 0.32	2.74 ± 0.40	1.46E-05
**hsa-miR-192**	3.75 ± 0.82	2.56 ± 0.30	2.49 ± 0.40	2.55 ± 0.49	4.51E-10
**hsa-miR-193a-3p**	5.25 ± 1.22	2.65 ± 0.49	2.75 ± 0.65	2.76 ± 0.53	0
**hsa-miR-194**	4.98 ± 1.53	3.33 ± 0.94	3.28 ± 1.04	3.09 ± 0.83	1.11E-05
**hsa-miR-19a**	3.10 ± 0.48	2.58 ± 0.36	2.55 ± 0.26	2.53 ± 0.32	6.38E-05
**hsa-miR-19b**	7.22 ± 1.16	4.99 ± 1.91	3.66 ± 1.49	4.33 ± 1.80	1.15E-06
**hsa-miR-200a-star**	4.16 ± 0.89	2.90 ± 0.54	2.75 ± 0.44	2.93 ± 0.45	3.10E-09
**hsa-miR-200a**	6.75 ± 1.87	4.10 ± 1.47	3.55 ± 1.49	3.68 ± 1.53	6.00E-07
**hsa-miR-203**	6.09 ± 2.68	3.97 ± 1.76	3.31 ± 1.25	3.26 ± 1.24	2.68E-05
**hsa-miR-21-star**	5.71 ± 1.41	3.58 ± 1.12	3.19 ± 1.09	3.28 ± 0.99	3.98E-08
**hsa-miR-21**	7.05 ± 2.00	4.86 ± 1.91	3.67 ± 1.50	4.79 ± 2.04	8.21E-05
**hsa-miR-214-star**	3.90 ± 1.38	2.62 ± 0.69	2.54 ± 0.47	2.61 ± 0.49	4.32E-06
**hsa-miR-22-star**	3.12 ± 0.63	2.55 ± 0.24	2.60 ± 0.31	2.49 ± 0.21	8.00E-06
**hsa-miR-22**	9.86 ± 0.92	6.35 ± 2.64	5.40 ± 2.83	6.34 ± 2.87	1.20E-04
**hsa-miR-24-2-star**	4.11 ± 1.00	2.96 ± 0.79	2.67 ± 0.42	2.83 ± 0.61	1.13E-06
**hsa-miR-27b-star**	3.06 ± 0.38	2.49 ± 0.39	2.49 ± 0.33	2.54 ± 0.34	1.00E-04
**hsa-miR-29b**	3.74 ± 1.31	2.40 ± 0.21	2.42 ± 0.23	2.47 ± 0.38	4.21E-09
**hsa-miR-29c-star**	3.66 ± 1.08	2.41 ± 0.32	2.33 ± 0.25	2.43 ± 0.27	6.70E-11
**hsa-miR-29c**	4.27 ± 1.48	2.56 ± 0.35	2.52 ± 0.28	2.55 ± 0.48	9.49E-11
**hsa-miR-30b**	6.59 ± 1.87	4.25 ± 1.57	3.82 ± 1.47	4.34 ± 1.76	1.44E-04
**hsa-miR-30c-2-star**	4.27 ± 1.22	3.23 ± 0.99	2.75 ± 0.61	2.98 ± 0.55	1.90E-05
**hsa-miR-30e**	4.95 ± 1.35	3.23 ± 0.81	2.68 ± 0.43	3.07 ± 0.72	3.36E-10
**hsa-miR-3160**	2.77 ± 0.41	2.47 ± 0.24	2.32 ± 0.23	2.44 ± 0.20	1.26E-04
**hsa-miR-3172**	6.25 ± 0.50	3.74 ± 1.34	3.58 ± 1.45	4.15 ± 1.79	1.48E-05
**hsa-miR-331-3p**	5.18 ± 0.68	3.98 ± 1.24	3.57 ± 1.11	3.49 ± 0.95	8.87E-05
**hsa-miR-339-3p**	6.00 ± 0.56	4.34 ± 1.39	3.75 ± 1.55	3.97 ± 1.47	1.59E-04
**hsa-miR-375**	9.66 ± 1.24	5.46 ± 2.70	5.39 ± 2.84	5.14 ± 2.79	2.16E-05
**hsa-miR-4317**	3.40 ± 0.38	2.74 ± 0.35	2.63 ± 0.31	2.61 ± 0.27	2.92E-09
**hsa-miR-451**	4.65 ± 1.10	3.32 ± 0.81	3.06 ± 0.72	3.47 ± 0.70	6.66E-06
**hsa-miR-455-5p**	2.99 ± 0.39	2.39 ± 0.27	2.44 ± 0.22	2.46 ± 0.20	6.54E-08
**hsa-miR-497**	6.92 ± 1.60	3.79 ± 1.46	3.50 ± 1.37	3.86 ± 1.49	2.62E-08
**hsa-miR-551b-star**	4.99 ± 0.95	4.12 ± 0.91	3.69 ± 0.99	4.96 ± 1.03	5.20E-05
**hsa-miR-590-3p**	2.04 ± 0.12	2.25 ± 0.15	2.36 ± 0.20	2.28 ± 0.22	1.86E-04
**hsa-miR-660**	4.29 ± 1.19	3.11 ± 0.46	2.91 ± 0.65	3.19 ± 0.73	1.88E-05
**hsa-miR-769-5p**	4.38 ± 0.72	2.75 ± 0.46	2.64 ± 0.46	2.67 ± 0.41	7.77E-16
**hsa-miR-887**	3.92 ± 1.18	2.78 ± 0.55	2.68 ± 0.49	2.85 ± 0.57	9.95E-06
**hsa-miR-98**	4.35 ± 0.87	3.22 ± 0.69	3.11 ± 0.79	2.98 ± 0.63	6.39E-06

fMBC: familial male breast cancer; fFBC: familial female breast cancer. Data are reported as mean log_2_[intensity values] ± standard deviation.

FBC1 included BRCA-related fFBCs (34.78%) and BRCAX fMBCs (52.17%); FBC2 included 61.1% BRCAX fFBC samples and FBC3 contained an equal number of BRCA-related and BRCAX fFBCs.

Our question was the identification of relationship between the results of sample clustering and the clinico-pathological data (ER, PgR and Her2), which are still considered the gold standard for diagnosis and prognosis of breast tumors.

Matching these results with all clinico-pathological features, a correlation between ER-status and the three sample clusters have been found. In detail, FBC1 samples are 82.6% ER-positive; FBC2 samples are mostly ER-negative (66.6%); indeed, FBC3 samples are ER-positive. The three clusters are distinct by different expression of two clusters of miRNAs, with a high correlation coefficient (*R*^*2*^ > 0.55). These clusters are CLU1 (*R*^*2*^ > 0.64) and CLU2 (*R*^*2*^ > 0.84), which included 17 and 36 miRNAs, respectively (Table 2). In particular, CLU1 is overexpressed in FBC1 and downregulated in FBC2. CLU2 showed an opposite behavior than CLU1 in FBC1 and FBC2. FBC3 has a not so clearly defined pattern of expression of CLU1 and CLU2 (Figure 2A).

**Table 2:** **MiRNAs belonging to CLU1 and CLU2 respectively, both evidenced by hierarchical clustering**

**CLU1**	**CLU2**
mir-106b	mir-1259
mir-1274a	mir-125a-3p
mir-1274b	mir-1271
mir-143	mir-128
mir-152	mir-140-5p
mir-15a	mir-143star
mir-19b	mir-148a
mir-200a	mir-148b
mir-203	mir-17star
mir-21 star	mir-181c-star
mir-21	mir-188-5p
mir-22	mir-192
mir-30b	mir-193a-3p
mir-3172	mir-194
mir-339-3p	mir-19a
mir-375	mir-200a-star
mir-497	mir-214star
	mir-22star
	mir-24-2-star
	mir-27b-star
	mir-29b
	mir-29c
	mir-30c-2star
	mir-30e
	mir-3160
	mir-331-3p
	mir-4317
	mir-451
	mir-455-5p
	mir-551b-star
	mir-590-3p
	mir-660
	mir-769-5p
	mir-887
	mir-98
	mir-29c-star

In conclusion, these results seemed to indicate that familial BCs could be stratified accordingly to the pattern of expression of CLU1 and CLU2.

### Triple negative BCs: BRCA-related and sporadic BC

Triple negative breast tumors (TNBCs) are characterized by a more aggressive phenotype and include subgroups with features shared with BRCA-related BCs. For this reason we explored the pattern of expression of miRNAs in 21 BRCA-related and 27 sporadic BCs, taking into account TNBC and NTNBC immunophenotype (Figure 1).

**Figure 3:**
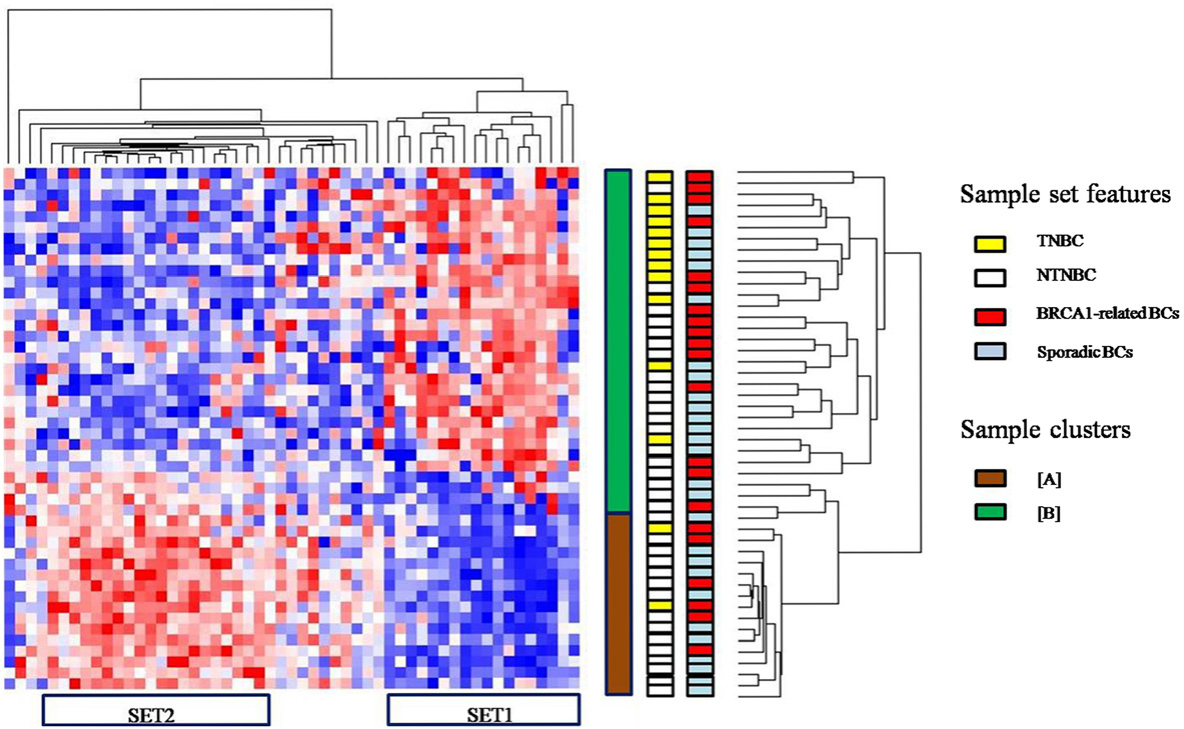
**Clustering analysis of BRCA1-related and sporadic breast tumors.** (Pink indicates overexpressed miRNAs; blue indicates underexpressed miRNAs).

Unsupervised hierarchical clustering of BRCA-related and sporadic BCs evidenced two sample clusters: one of them [A] included 64.7% sporadic BCs which are triple positive; interestingly, the second [B] is separated into two subclusters, as shown in the dendrogram, one of 83.33% TNBCs and the second of 89.47% NTNBCs, almost including an equal number of BRCA-related and sporadic samples (Figure 3). [A] and [B] are characterized by different expression of two clusters of miRNAs: SET1 (*R*^*2*^ > 0.75) and SET2 (*R*^*2*^ > 0.87) (Table 3). In detail, SET1, consisting of 18 miRNAs mainly included in CLU1 plus miR-125a-3p and miR-331-3p, is overexpressed in the cluster including TN and NTN ([B]). Sample cluster of triple positive sporadic BCs ([A]) overexpressed SET2, consisting of 21 miRNAs which are a subgroups of CLU2.

**Table 3:** **MiRNAs belonging to SET1 and SET2 respectively, both evidenced by hierarchical clustering**

**SET1**	**SET2**
mir-203	mir-214star
mir-106b	mir-188-5p
mir-143	mir-200a-star
mir-22	mir-193a-3p
mir-152	mir-143star
mir-497	mir-27b-star
mir-21	mir-19a
mir-30b	mir-140-5p
mir-19b	mir-148b
mir-200a	mir-29b
mir-15a	mir-181c-star
mir-339-3p	mir-3160
mir-21 star	mir-22star
mir-125a-3p	mir-4317
mir-331-3p	mir-1259
	mir-590-3p
	mir-455-5p
	mir-29c-star
	mir-769-5p
	mir-29c
	mir-887

### GO functional annotation of miRNA clusters

To better understand the biological meaning of the previously evidenced clusters of miRNAs, we performed a pathway enrichment analysis. Briefly, databases of experimentally validated targets of miRNAs have been queried (TarBase, miRtarbase and miRecords) in order to achieve a “validated” functional understanding.

#### Pathway enrichment analysis of CLU1 and CLU2

CLU1, which is overexpressed in familial sample cluster FBC1, indicated an association to GO functional categories related to “epithelial to mesenchimal transition”, “hypoxia”, “angiogenesis”, “regulation of cell death”, “cell motility”, “cell cycle” and, interestingly, to the “response to estrogen stimulus”. GO functional categories related to CLU2, which is overexpressed in FBC2, are, in particular, “transcription”, “regulation of transcription from RNA polymerase II promoter”, “transcription repressor activity”, “negative regulation of gene expression”, “methylation“, “histone acetyltransferase activity” and “lysine N-acetyltransferase activity” (Table 4).

**Table 4:** **Signaling pathways associated to CLU1 and CLU2 clusters**

**Signaling pathway**	**P-value**	**Cluster**
Regulation of cell death	3.80E-09	CLU1
Cell cycle	7.00E-08	CLU1
ER-nuclear signaling pathway	4.10E-06	CLU1
Angiogenesis	2.00E-04	CLU1
Response to estrogen stimulus	7.30E-04	CLU1
Epithelial to mesenchymal transition	3.40E-03	CLU1
Ras protein signal transduction	9.80E-03	CLU1
Cell motility	1.20E-02	CLU1
Response to hypoxia	1.30E-02	CLU1
Transcription	9.50E-06	CLU2
Regulation of transcription from RNA polymerase II promoter	1.20E-05	CLU2
Transcription repressor activity	1.30E-05	CLU2
Negative regulation of gene expression	3.70E-05	CLU2
Methylation	2.40E-04	CLU2
Histone acetyltransferase activity	1.60E-03	CLU2
Lysine N-acetyltransferase activity	1.60E-03	CLU2

Such result seems to indicate that CLU1 is responsible for regulation of cellular pathways and CLU2 is involved in epigenetic activities.

#### Pathway enrichment analysis of SET1 and SET2

GO term enrichment analysis of SET1 and SET2 revealed, as expected, no particular differences compared to results regarding CLU1 and CLU2. Interestingly, the term “ER nuclear signaling pathway” merged from the analysis for SET1, indicating a peculiar role for the estrogen receptor, and the term “histone modification” was found, which did not merged from the functional annotation analysis for CLU1.

In conclusion, the analysis did not give us results different from those of CLU1 and CLU2 because SET1 and SET2 are not so different in their miRNA content.

## Discussion

Since miRNAs deregulation was initially described in BC [25], numerous studies have been focused on the most differentially expressed miRNAs allowing to a better knowledge about their biological role in this heterogeneous disease. Although increasing efforts have been undertaken to elucidate the potential use of miRNAs as diagnostic and prognostic tool, the informative power of miRNA profiles in breast tumor still remains unclear. There is an extensive number of studies investigating the expression of miRNAs in breast carcinoma but few data are available when the focus is restricted to specific breast tumor subgroups such as familial BCs. Our aim was to identify a specific miRNA signature in familial BC subgroup according to clinico-pathological parameters, also considering the sporadic BC. Until recently, there is a limited number of reports exploring miRNA profile in familial breast tumors, which considered only the family history and the mutational status of BRCA [9-12]. In this regard, this is the first study evaluating whether there is an epigenetic regulation able to highlight a specific clinico-pathological assessment in familial and sporadic breast tumors.

Our hierarchical analysis highlighted two different clusters, CLU1 and CLU2, consisting of 17 and 36 miRNAs, respectively. Considering FBC1 and FBC2, CLU1 and CLU2 were able not only to discriminate ER-negative from ER-positive breast tumors but also female BRCA-unrelated, female BRCA-related and male BCs. A key role for the estrogen receptor alpha (ERα) in the pathogenesis of BC has been well described. ERα is correlated to survival and cell proliferation pathways through both genomic and non-genomic mechanisms, which in turn can interact with each other in crosstalk [26]. Our enrichment analysis highlighted numerous signaling pathways specifically associated with both CLU1 and CLU2. Among these, cellular signals such as hypoxic, epithelial to mesenchymal transition (EMT) and Ras protein signals transduction involved in the tumor progression were mainly correlated with CLU1, whereas chromosomal regulation and epigenetic mechanisms were found mainly associated with CLU2. Since an over-expression of CLU1 was observed in female BRCA-related and male breast tumors expressing ER, we supposed that ER signaling pathways could be more involved in the pathogenesis of this BC subgroups. To support our hypothesis, ER-nuclear signaling pathway and response to estrogen stimulus were found among the pathways associated with CLU-1 miRNAs co-expression. On the contrary, the over-expression of CLU2 in ER-negative familial subgroup highlighted the potential role of epigenetic mechanisms in the regulation of ER expression. A set of microRNAs such as mir-342, mir-299, mir-217, mir-190, mir-135b and mir-218 were found to be associated with the estrogen receptor status in breast tumor samples in which a miRNA signature able to predict ER, progesterone receptor (PgR) and human epidermal receptor-2 (HER2) status was highlighted through an artificial neural network (ANN) analysis [27]. Recently, the association between miRNAs and estrogen receptor has been also investigated by numerous studies exploring the epigenetic role in the endocrine resistance [28]. Among the mechanisms involved in the acquisition of a non-responsive phenotype, the post-transcriptional regulation of ER by miRNAs has been revealed. In this regard mir-22, mir-206, mir-222, mir-221 and mir-18a have been suggested to target ER, inducing an estrogen signaling reduction [29]. Among CLU2-signaling pathways revealed by our enrichment analysis, histone acetyltransferase and a lysine N-acetyltransferase activities were reported. Interestingly, H3 and H4 histones deacetylation by HDAC is one of the mechanism described for the ER promoter activity regulation [30]. Moreover, our analysis revealed an association of the transcription cofactor activity with CLU-2 miRNAs coexpression, supporting the hypothesis of an ER transcriptional activity influenced by changes in coregulatory protein expression levels previously described [26]. In fact, a key role for miR-17-5p in ER coactivator SRC-3/AIB1/NCOA3 regulation was reported [31].

Among the diverse subgroups in which the BC is subdivided, TNs account for 10%-15% of all BCs. TNBCs are used to define BC that lacks hormone receptors and HER2 and they represent the most aggressive subtype with a poor prognosis [32]. According to Lehmann classification, TNBCs are subdivided in six different subtypes such as basal I and II, mesenchymal and mesenchymal stem cell-like, immunomodulatory and androgen pathway enriched [33]. Over 80% of hereditary BRCA1-mutated cancers are TNBCs and the pivotal role played by the inactivation of BRCA1 in TNBC immunophenotype has been suggested by several studies investigating the similar clinical outcomes and histological characteristics between hereditary BRCA1-related and sporadic TNBC [34]. Stratifying BC in triple- and non triple negative tumors, two miRNA clusters, SET-1 and SET-2, were delineated by our hierarchical analysis. SET-1 included almost all CLU-1 miRNAs with the exception of mir-125a and mir-331, whereas SET-2 consisting of several CLU-2 miRNAs. Different expression of both SET-1 and SET-2 delineated two groups: SET-1 over-expressing group [B] that included BRCA-related and sporadic breast tumors with and without TN phenotype and SET-2 over-expressing group [A], consisting mainly of sporadic NTNBCs. Surprisingly, within [B] a sub-hierarchical cluster able to discriminate TN from NTN breast tumors was observed. According to miRNA profile, the characteristic tendency of TNBCs to cluster differentially compared to other breast tumor groups expressing ER, PgR or HER2, was reported in a set of normal, DCIS and invasive BC cases [35]. Furthermore, a more recent study focused on TNBC and normal tissues highlighted a profile of 116 deregulated miRNAs, among which let-7b, let-7c, mir-126, mir-145 and mir-205 were the most down-modulated and the cluster mir-17/92, mir-106b, mir-8 family, mir-21 and mir-155 were the most up-modulated [5]. Interestingly, in our study, both TNBC and NTNBC subgroups included either BRCA-related as sporadic breast tumors. BRCA1 is a tumor suppressor gene that plays a pivotal role in the maintenance of genomic stability. The presence of germline mutation in BRCA1 gene and the loss of protein function increases the risk of BC development [36]. It has been well-reported that sporadic basal tumors, show reduced BRCA1 mRNA expression, frequently due to an epigenetic modification of the BRCA1 gene [14,37]. In the last years, the concept of ‘BRCAness’ was postulated in order to identify a significant proportion of sporadic BCs with BRCA-like functional abnormalities and characterized by an analogous BRCA1 treatment susceptibility [38]. Interestingly our data, in according to miRNA profile, highlighted a fraction of NTNBCs, consisting of both BRCA-related and sporadic breast tumors that clustered with TNBCs. Differently from [A] group consisting of 88.23% of sporadic BCs, the fraction of NTNBC belonged to [B] group, included both sporadic and BRCA-related breast tumors. However, our data needed of further analysis in greater BC cohort. Accordingly to literature data, we found that BRCA-related and sporadic TNBC clustered together supporting the hypothesis of a similar epigenetic regulation in these tumors.

## Conclusions

Our results highlighted a key role for BRCA1/2 genes and ER in familial BC pathogenesis. BRCA1 and ER crosstalk has already been investigated, describing a dual role for BRCA1 as both co-repressor and co-activator of ER-mediated transcription [29]. In conclusion, miRNAs expression pattern in familial and sporadic BCs, related to immunophenotype, could better clarify similarities and differences between these two groups.

## Abbreviations

fBC: Familial breast cancer
fFBC: Familial female breast cancer
fMBC: Familial male breast cancer
GO: Gene ontology
TNBC: Triple negative breast cancer
NTNBC: Not triple negative breast cancer
ER: Estrogen receptor
PR: Progesteron receptor
